# Supplementary material for: Direct binding of arsenicals to nuclear transport factors disrupts nucleocytoplasmic transport
Source: bioRxiv. 2025 Jan 15:2025.01.13.632748. Preprint. [Version 1] doi: 10.1101/2025.01.13.632748 (PMC11761705; doi:10.1101/2025.01.13.632748)
Supplement: Supplementary file 1 [file NIHPP2025.01.13.632748V1-supplement-1.pdf]

bioRxiv preprint doi: https://doi.org/10.1101/2025.01.13.632748; this version posted January 15, 2025. The copyright holder for this preprint (which was not certified by peer review) is the author/funder, who has granted bioRxiv a license to display the preprint in perpetuity. It is made available under aCC-BY 4.0 International license.

SUPPLEMENTARY MATERIAL

Table S1. All proteins detected by LC/MS/MS (data file).

Table S2. List of 174 candidate arsenic-binding proteins.

Table S3. List of strains and plasmids used.

Table S4. Datasets used for comparisons.

Figure S1. Integration of chemical-genetic and genetic interaction data to identify *bona fide* arsenic toxicity targets. Negative genetic interactors (including negative genetic, synthetic growth defect, synthetic lethality) of selected arsenic-binding hits were retrieved from SGD (Wong et al., 2023) and compared to a compendium of 712 As(III) sensitive *S. cerevisiae* mutants that contains the genes identified at least once in four genome-wide phenotypic screens (Haugen et al., 2004; Pan et al., 2010; Thorsen et al., 2009; Zhou et al., 2009). The significance of the overlaps between the datasets (negative genetic interactor sets and As(III) sensitive set) was calculated by the hyper-geometric test and the corresponding *P*-values are indicated.

Figure S2. AlphaFold structure predictions and cysteine mapping for Srp1, Kap95, Kap121/Pse1, Crm1, Kap123, Sxm1/Kap108, Msn5, Nup84, and Nup188. Distances between pairs of adjacent or proximal cysteines are indicated. The structure predictions are based on experimental crystal structure data for all proteins except for Kap123, Kap108/Sxm1 and Msn5.

Figure S3. Mislocalization of Srp1 in As(III)-exposed cells.

S3A. Quantification of Srp1-GFP nuclear envelope (NE) localization (left panel) and foci formation (right panel) in the presence and absence of 1.5 mM As(III) and/or 0.2 mg/ml cycloheximide (CHX). Srp1–GFP distribution was scored by fluorescence microscopy and quantified by visual inspection. The bars represent the mean ± SD of three independent biological repeats of a total of 300 cells. Significance was calculated using un-paired two-tailed student's t-test with either the untreated control (for just As(III)-exposure) or CHX (for CHX+As(III) treated cells) as the comparison, and *P*-values are according to: ** > 0.01, *** > 0.001.

S3B. Cells were exposed to 1.5 mM As(III) for 1 h, then washed twice and resuspended in medium without As(III) in the presence or absence of 0.2 mg/ml CHX. Srp1–GFP distribution was scored by fluorescence microscopy and quantified as in S3A. Significance was calculated using un-paired two-

bioRxiv preprint doi: https://doi.org/10.1101/2025.01.13.632748; this version posted January 15, 2025. The copyright holder for this preprint (which was not certified by peer review) is the author/funder, who has granted bioRxiv a license to display the preprint in perpetuity. It is made available under aCC-BY 4.0 International license.

tailed student's t-test of three independent biological replicates, with 1 h As(III)-exposed cells as the control sample. *P*-values according to * > 0.05, ** > 0.01, *** > 0.001.

Figure S4. Nup localization and levels during As(III) stress.

S4A. Cycloheximide (CHX) does not prevent Nup84-GFP mislocalization in As(III)-stressed cells. Quantification of Nup84-GFP foci formation in the presence or absence of 1.5 mM As(III) and 0.2 mg/ml CHX. Foci formation was detected with fluorescence microscopy and quantified by visual inspection. The bars represent the mean ± SD of three independent biological repeats of a total of 300 cells. Significance was calculated using un-paired two-tailed student's t-test with CHX-treated cells as the comparison, and P-values are according to: ** > 0.01, *** > 0.001.

S4B. Nup84-GFP and Nup188-GFP protein levels are largely unaffected by As(III). Western blot of the total lysate from cells expressing Nup84-GFP or Nup188-GFP in the absence (control) and presence of As(III). The lower panel shows the corresponding SDS-PAGE gels as loading controls. The images shown are representative of at least two biological repeats.

Figure S5. Nuclear transport is unaffected during short-term As(III) exposure.

Cells expressing GFP-tagged versions of the transcription factors Yap1, Msn2, and Sfp1 were either left untreated (control) or exposed to the indicated concentrations of As(III), and their localization determined by fluorescence microscopy. Quantification was done by visual inspection and the bars represent the mean ± SD of three independent biological repeats of a total of 300 cells. Significance was calculated using un-paired two-tailed student's t-test with the untreated control at the respective time point as the comparison, and *P*-values are according to: *> 0.05, ** > 0.01, *** > 0.001.

S1.

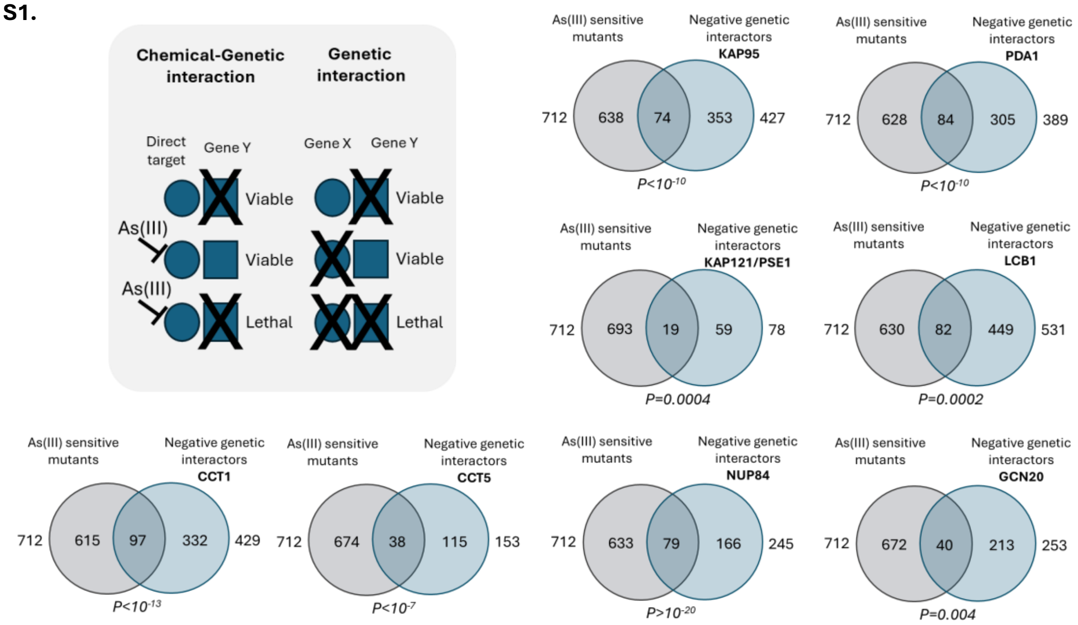
Chemical-Genetic interaction
Genetic interaction
Direct target
Gene Y
Gene X
Gene Y
Viable
Viable
As(III)
Viable
Viable
As(III)
Lethal
Lethal
As(III) sensitive mutants
Negative genetic interactors KAP95
712
638
74
353
427
P<10-10
As(III) sensitive mutants
Negative genetic interactors PDA1
712
628
84
305
389
P<10-10
As(III) sensitive mutants
Negative genetic interactors KAP121/PSE1
712
693
19
59
78
P=0.0004
As(III) sensitive mutants
Negative genetic interactors LCB1
712
630
82
449
531
P=0.0002
As(III) sensitive mutants
Negative genetic interactors CCT1
712
615
97
332
429
P<10-13
As(III) sensitive mutants
Negative genetic interactors CCT5
712
674
38
115
153
P<10-7
As(III) sensitive mutants
Negative genetic interactors NUP84
712
633
79
166
245
P>10-20
As(III) sensitive mutants
Negative genetic interactors GCN20
712
672
40
213
253
P=0.004

S2.

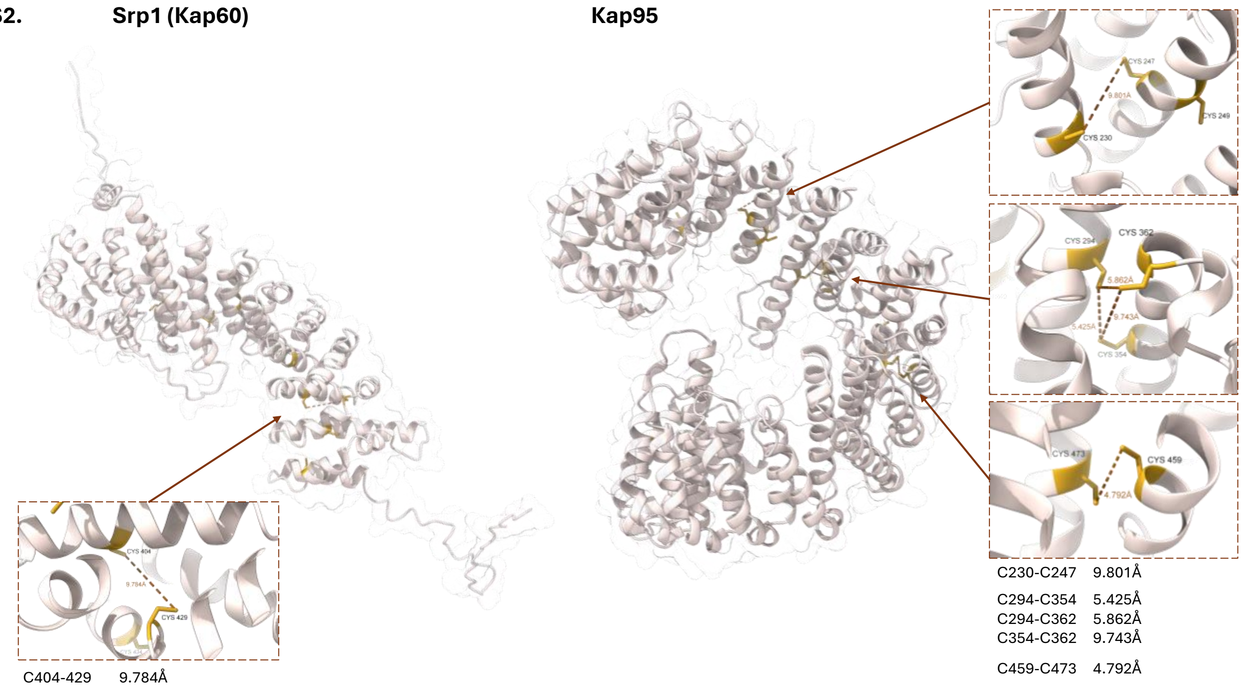

Srp1 (Kap60)
Kap95
C404-429 9.784Å
C230-C247 9.801Å
C294-C354 5.425Å
C294-C362 5.862Å
C354-C362 9.743Å
C459-C473 4.792Å

S2.

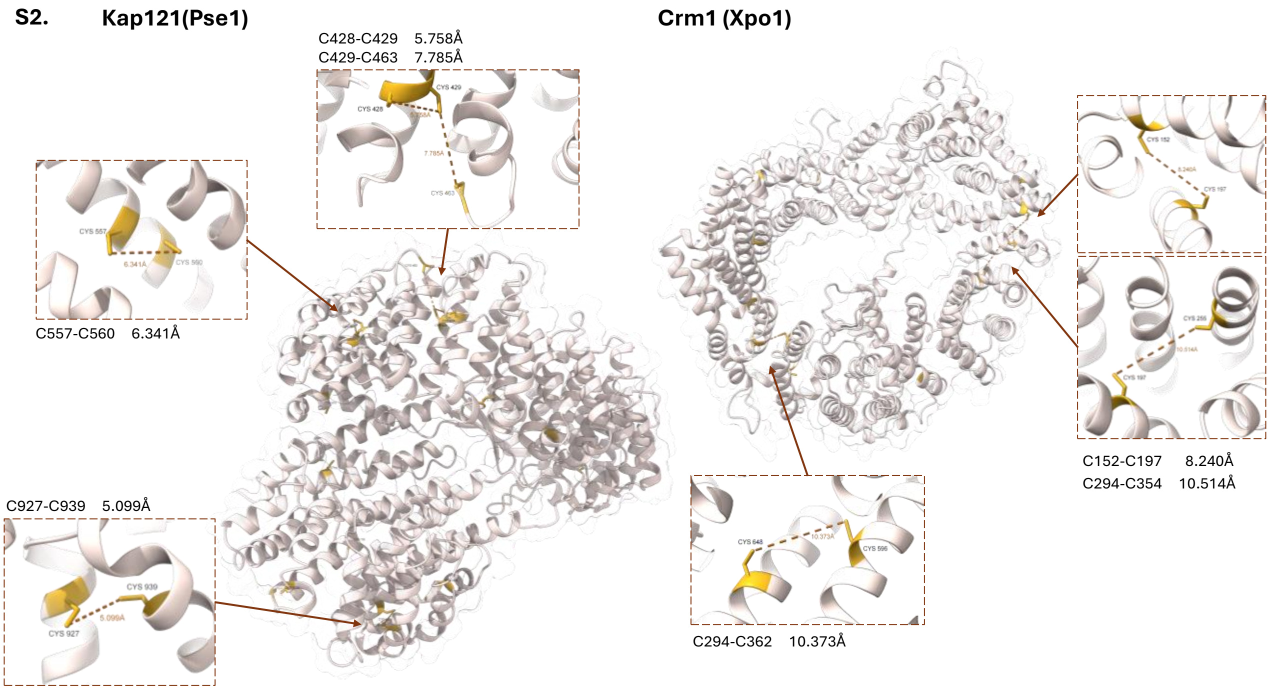

Kap121(Pse1)
Crm1 (Xpo1)
C428-C429 5.758Å
C429-C463 7.785Å
C557-C560 6.341Å
C927-C939 5.099Å
C152-C197 8.240Å
C294-C354 10.514Å
C294-C362 10.373Å

S2. **Kap123** **Kap108(Sxm1)**

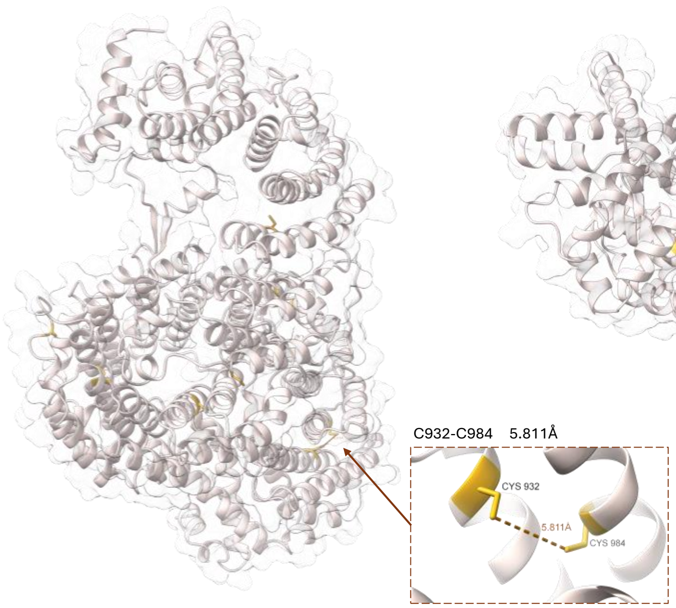

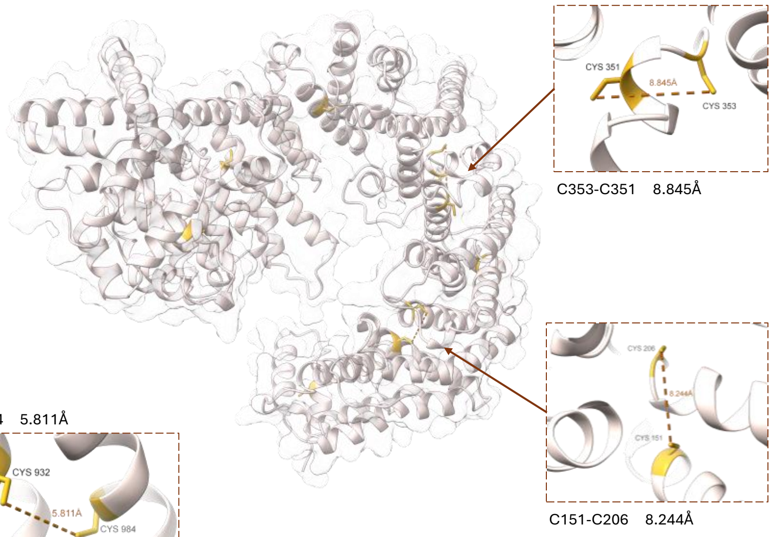

S2. **Msn5**

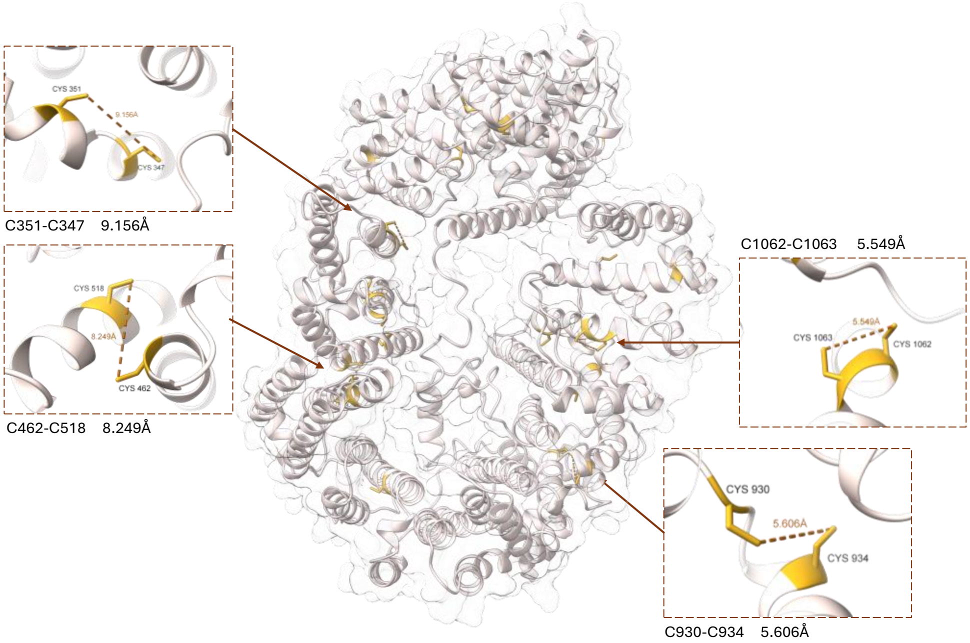

S2.

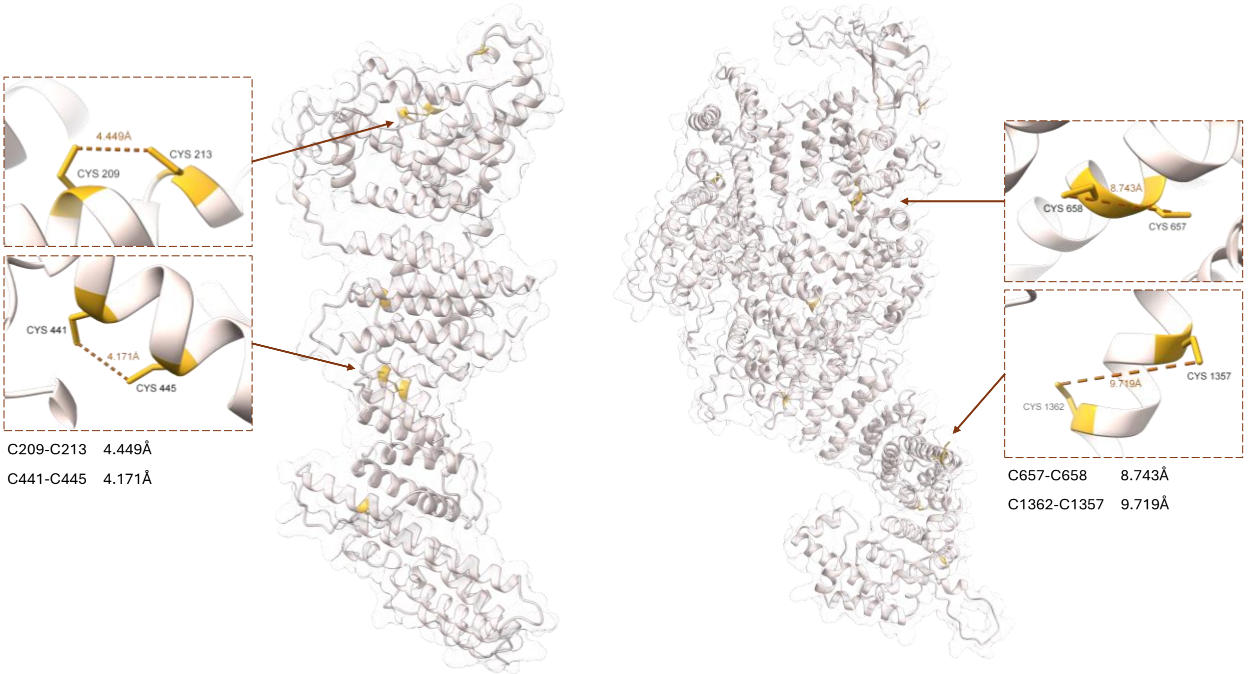

Nup84
Nup188
4.449Å
CYS 209
CYS 213
CYS 441
4.171Å
CYS 445
C209-C213 4.449Å
C441-C445 4.171Å
8.743Å
CYS 658
CYS 657
9.719Å
CYS 1357
CYS 1362
C657-C658 8.743Å
C1362-C1357 9.719Å

S3A.

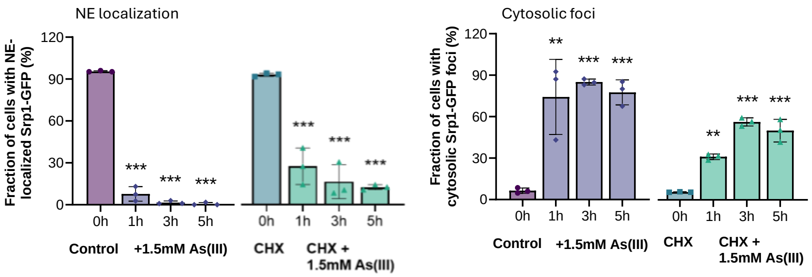

NE localization
Fraction of cells with NE-localized Srp1-GFP (%)
120
90
60
30
0
0h 1h 3h 5h 0h 1h 3h 5h
Control +1.5mM As(III) CHX CHX + 1.5mM As(III)
Cytosolic foci
Fraction of cells with cytosolic Srp1-GFP foci (%)
0h 1h 3h 5h 0h 1h 3h 5h
Control +1.5mM As(III) CHX CHX + 1.5mM As(III)

S3B.

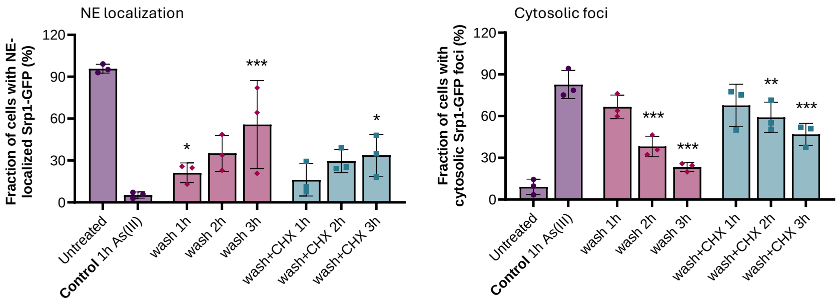

NE localization
Fraction of cells with NE-localized Srp1-GFP (%)
120
90
60
30
0
Untreated
Control 1h As(III)
wash 1h
wash 2h
wash 3h
wash+CHX 1h
wash+CHX 2h
wash+CHX 3h
Cytosolic foci
Fraction of cells with cytosolic Srp1-GFP foci (%)

S4A.

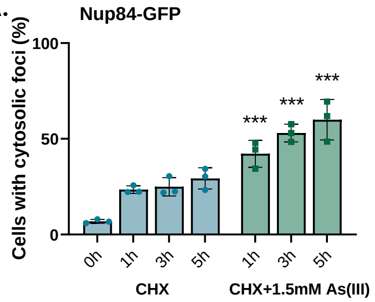

Nup84-GFP
Cells with cytosolic foci (%)
100
50
0
***
***
***
0h
1h
3h
5h
1h
3h
5h
CHX
CHX+1.5mM As(III)

S4B.

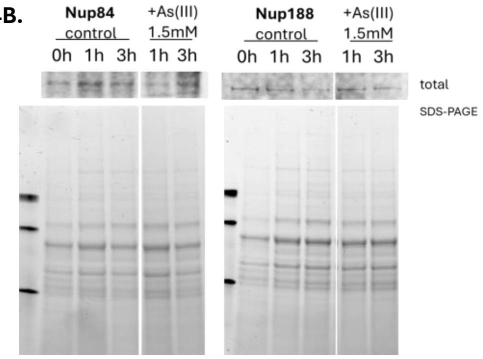

Nup84
control
+As(III)
1.5mM
0h 1h 3h 1h 3h
Nup188
control
+As(III)
1.5mM
0h 1h 3h 1h 3h
total
SDS-PAGE

S5.

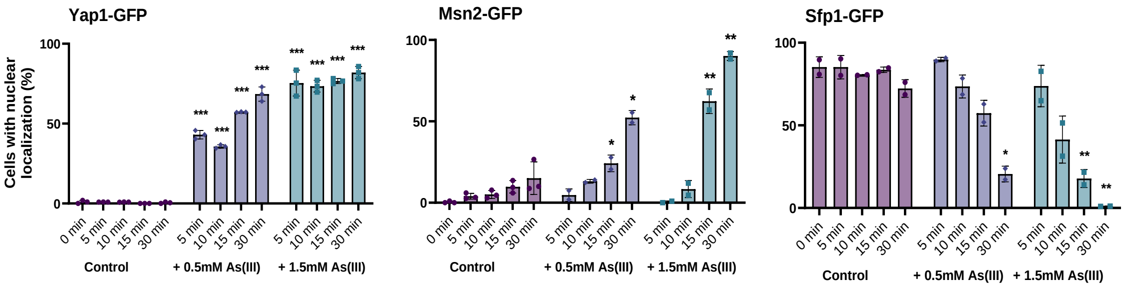

Yap1-GFP
Msn2-GFP
Sfp1-GFP
Cells with nuclear localization (%)
100
50
0
0 min
5 min
10 min
15 min
30 min
Control
+ 0.5mM As(III)
+ 1.5mM As(III)
***
**
*
